# Viral dominance of reassortants between canine influenza H3N2 and pandemic (2009) H1N1 viruses from a naturally co-infected dog

**DOI:** 10.1186/s12985-015-0343-z

**Published:** 2015-09-04

**Authors:** Woonsung Na, Kwang-Soo Lyoo, Eun-jung Song, Minki Hong, Minjoo Yeom, Hyoungjoon Moon, Bo-Kyu Kang, Doo-Jin Kim, Jeong-Ki Kim, Daesub Song

**Affiliations:** Korea Research Institute of Bioscience and Biotechnology, Daejeon, Republic of Korea; Korea Zoonosis Research Institute, Chonbuk National University, Iksan, Republic of Korea; Department of Pharmacy, College of Pharmacy, Korea University, Sejong, Republic of Korea; Green Cross Veterinary Products, Yong-In, Republic of Korea

**Keywords:** Influenza virus, Canine H3N2, Pandemic H1N1, Genetic dominance, Reassortment

## Abstract

**Background:**

Since avian-origin H3N2 canine influenza virus (CIV) was first identified in South Korea in 2008, the novel influenza virus has been reported in several countries in Asia. Reverse zoonotic transmission of pandemic H1N1 (2009) influenza virus (pH1N1) has been observed in a broad range of animal species. Viral dominance and characterization of the reassortants of both viruses was undertaken in the present study.

**Findings:**

Here we describe the viral dominance of 23 CIV reassortants between pH1N1 and canine H3N2 influenza viruses from a naturally co-infected dog. These results indicate that the M gene of pandemic H1N1 and the HA gene of canine H3N2 are predominant in the reassortants. Furthermore, unlike the original canine H3N2 virus, some reassortants showed high pathogenicity in mice.

**Conclusions:**

This study suggests that continuous monitoring of influenza infection in companion animals may be necessary to investigate the potential of the emergence of novel influenza viruses.

## Findings

Outbreaks of the infections caused by canine H3N2 (cH3N2) virus of avian origin, which causes severe respiratory symptoms in dog populations, have been continuously reported in South Korea, China, and Thailand since 2007, and inter–mammalian species transmission (dog to cat) has also been reported [1–4]. In August 2010, the World Health Organization announced that pandemic H1N1 (pH1N1) had moved into the post-pandemic period, although localized outbreaks of various magnitudes continue to occur. In particular, pH1N1 has been transmitted from humans to several animal species (termed “reverse zoonoses”), and many novel reassortant viruses have been generated [5–9]. Although most cases of reverse zoonoses have been reported in the pig population, the primary companion animals could be co-infected with pH1N1 and cH3N2 viruses due to their ecophysiological characteristics (synanthropicity). In this study, we isolated 23 distinct viral genotypes of influenza reassortants by using a nasal swab of a co-infected dog and characterized the genotypes of the reassortants.

From 2010 to 2012 in South Korea, we collected 213 nasal swabs from sick dogs showing clinical respiratory signs and tested the presence of canine influenza virus (CIV). Mixed infection with cH3N2 and pH1N1 influenza subtypes was confirmed in an individual dog (a swab specimen) using a commercial real-time reverse transcription–polymerase chain reaction (RT-PCR) kit (SensiFAST™; Bioline Inc.,Taunton, MA, USA). The real time RT-PCR targeted specific sequences of HA and NA genes for pH1N1 or cH3N2 influenza viruses. To determine the unidentified features, we performed single-plaque purification assays using the swab, and then the viruses were plaque-purified in MDCK cells.

Genotyping of plaque-purified viruses were performed as previously described by Hoffman et al. (2001) with slight modification [10]. Briefly, one-step RT-PCR (Qiagen) was conducted using universal primers, and each eight gene; polymerase basic 2 (PB2), polymerase basic 1 (PB1), polymerase acidic (PA), hemagglutinin (HA), nucleoprotein (NP), neuraminidase (NA), matrix (M), and nonstructural (NS), were sequenced with the amplified and purified gene segments.

In total 97 different plaques were purified, and then genotypes of the viruses were established based on the eight gene segments (PB2, PB1, PA, HA, NP, NA, M, and NS) that originated from pH1N1 or cH3N2 virus. The plaques were classified into 23 different genotypic groups (Table 1). Of the 23 identified genotypic patterns, three genotypes, namely, VC7 (~33 % of the clones contained the M gene from pH1N1; and the other genes were from cH3N2), VC78 (~12.4 % of the clones contained the M and NS genes from pH1N1; and the other genes were from cH3N2, and VC1235678 (~12.4 % of the clones contained the HA gene from cH3N2; and the other genes were from pH1N1) were found to be the most frequently observed among the 97 isolates based on the plaques. Only four clones (~4.1 %) contained all eight gene segments of cH3N2 (VCnone), while no clone containing all gene segments from pH1N1 was detected. These results indicate that an ecologically significant yield (93/97; 95.9 %) of the clones could be produced by recombination between cH3N2 and pH1N1. Additionally, the M and PA genes of pH1N1 were dominant in the reassortants between the pH1N1 and cH3N2 viruses (22 and 15 genotypes, respectively). Furthermore, 14 genotypes included the NS gene of pH1N1; ten included the PB1; nine included the PB2; eight included the NP; and eight included the NA. However, we did not detect any genotype pattern that included the HA gene of pH1N1 (Table 1). Overall, these results indicate that the M gene of pH1N1 and the HA gene of cH3N2 show genetic dominance in the reassortants between the pH1N1 and cH3N2 viruses.

**Table 1:** Genetic dominance among the 23 genotypes that were reassorted with canine H3N2 and pandemic (2009) H1N1 influenza viruses

Genotype^a^	Influenza gene segments								No. of isolates (%)
	PB2	PB1	PA	HA	NP	NA	M	NS	
VC7	C	C	C	C	C	C	P	C	32 (32.99)
VC78	C	C	C	C	C	C	P	P	12 (12.37)
VC1235678	P	P	P	C	P	P	P	P	12 (12.37)
VC235678	C	P	P	C	P	P	P	P	6 (6.19)
VC27	C	P	C	C	C	C	P	C	5 (5.15)
VCnone	C	C	C	C	C	C	C	C	4 (4.12)
VC378	C	C	P	C	C	C	P	P	4 (4.12)
VC37	C	C	P	C	C	C	P	C	3 (3.09)
VC123578	P	P	P	C	P	C	P	P	3 (3.09)
VC23578	C	P	P	C	P	C	P	P	2 (2.06)
VC678	C	C	C	C	C	P	P	P	2 (2.06)
VC12367	P	P	P	C	C	P	P	C	1 (1.03)
VC123678	P	P	P	C	C	P	P	P	1 (1.03)
VC12378	P	P	P	C	C	C	P	P	1 (1.03)
VC125678	P	P	C	C	P	P	P	P	1 (1.03)
VC13578	P	C	P	C	C	C	P	P	1 (1.03)
VC137	P	C	P	C	C	C	P	C	1 (1.03)
VC1378	P	C	P	C	C	C	P	P	1 (1.03)
VC237	C	P	P	C	C	C	P	C	1 (1.03)
VC3578	C	C	P	C	P	C	P	P	1 (1.03)
VC367	C	C	P	C	C	P	P	C	1 (1.03)
VC578	C	C	C	C	P	C	P	P	1 (1.03)
VC67	C	C	C	C	C	P	P	C	1 (1.03)

^a^Inserted gene segments of pH1N1 were labeled as VC#### (e.g., VC37 refers to the PA(3) and M(7) genes from pH1N1; the other genes are from cH3N2)

*C* canine H3N2 influenza virus; *P* pandemic H1N1 influenza virus

**Fig. 1:**
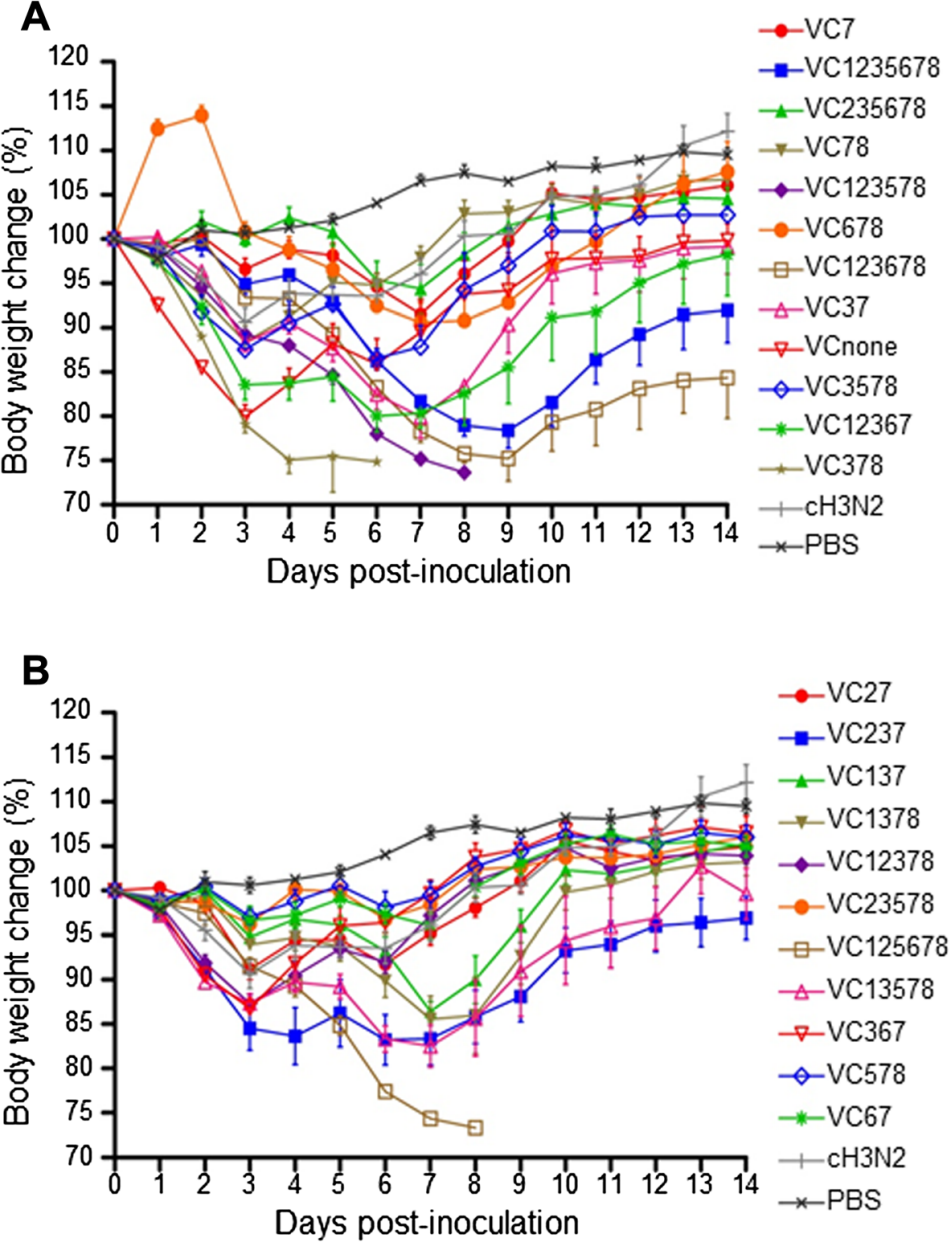
Body weight changes in mice. Mice (*n* = 6 per group) were inoculated intranasally with 10^6.5^ EID_50_ of each virus or PBS and monitored daily for 14 days for changes in body weight. Panel **a** shows body weight changes of groups of mice infected with VC7, VC1235678, VC235678, VC78, VC123578, VC678, VC123678, VC37, VCnone, VC3578, VC12367 or VC378. Panel **b** reveals those of groups of mice infected with VC27, VC237, VC137, VC1378, VC12378, VC23578, VC125678, VC13578, VC367, VC578 or VC67. The changes are represented as the percentage of weight on the day of inoculation (day zero), and the average of each group is shown

To examine the in vitro replicability and pathogenicity of a representative clone from each group, we determined HA titers and infectious and lethal doses. HA titers were measured using chicken red blood cells. The infective dose was measured using MDCK cells or embryonated chicken eggs and is presented as the 50 % tissue culture infectious dose (TCID_50_) or the 50 % egg infectious dose (EID_50_). The lethal dose was determined using mice and is presented as the 50 % mouse lethal dose (MLD_50_). The infective and lethal doses were calculated using the Reed-Muench method [11]. The clones examined replicated relatively well in mammalian cells and chicken embryos, as indicated by the HA titers, TCID_50_, and EID_50_, which were within the range of 2^8^–2^11^, 10^5.8^–10^7.8^, and 10^6.5^–10^8.75^, respectively (Table 2). However, these clones displayed a broad spectrum of changes in body weight and pathogenicity (asymptomatic to severe) in a mouse model (Fig. 1).

**Table 2:** Viral characteristics of the 23 genotypes in vivo and in vitro

Genotype	Subtype	Replicability and pathogenicity			
		HA titer	TCID_50_/mL	EID_50_/mL	MLD_50_
VC7	H3N2	2^10^	10^6.6^	10^8.5^	n.d.
VC1235678	H3N1	2^9^	10^5.9^	10^7.25^	10^7.25^
VC235678	H3N1	2^10^	10^5.8^	10^6.5^	n.d.
VC78	H3N2	2^11^	10^6.6^	10^7.5^	n.d.
VC123578	H3N2	2^10^	10^6.8^	10^7.5^	10^4.2^
VC678	H3N1	2^9^	10^6.6^	10^8^	n.d.
VC123678	H3N1	2^10^	10^6.4^	10^7.5^	10^4.9^
VC37	H3N2	2^11^	10^7.8^	10^8^	10^7^
VCnone	H3N2	2^10^	10^6.8^	10^6.5^	n.d.
VC3578	H3N2	2^10^	10^6.8^	10^7.5^	n.d.
VC12367	H3N1	2^10^	10^7^	10^7.75^	10^5.5^
VC378	H3N2	2^9^	10^7.5^	10^8^	10^4.25^
VC27	H3N2	2^10^	10^5.8^	10^8^	n.d.
VC237	H3N2	2^10^	10^6.8^	10^7.5^	10^7.26^
VC137	H3N2	2^9^	10^6.8^	10^8.5^	n.d.
VC1378	H3N2	2^9^	10^7.2^	10^8.25^	10^7.75^
VC12378	H3N2	2^10^	10^6.8^	10^7.5^	n.d.
VC23578	H3N2	2^9^	10^6.4^	10^7.25^	n.d.
VC125678	H3N1	2^8^	10^6.2^	10^6.5^	10^4^
VC13578	H3N2	2^9^	10^6.9^	10^7.25^	10^6.85^
VC367	H3N1	2^10^	10^6.6^	10^7.5^	n.d.
VC578	H3N2	2^10^	10^6.6^	10^8.75^	n.d.
VC67	H3N1	2^8^	10^6.9^	10^8.5^	n.d.

*EID*_*50*_ 50 % egg infectious dose; *HA* hemagglutinin; *MLD*_*50*_ 50 % mouse lethal dose; *n.d.* not determined; *TCID*_*50*_ 50 % tissue culture infectious dose

**Fig. 2:**
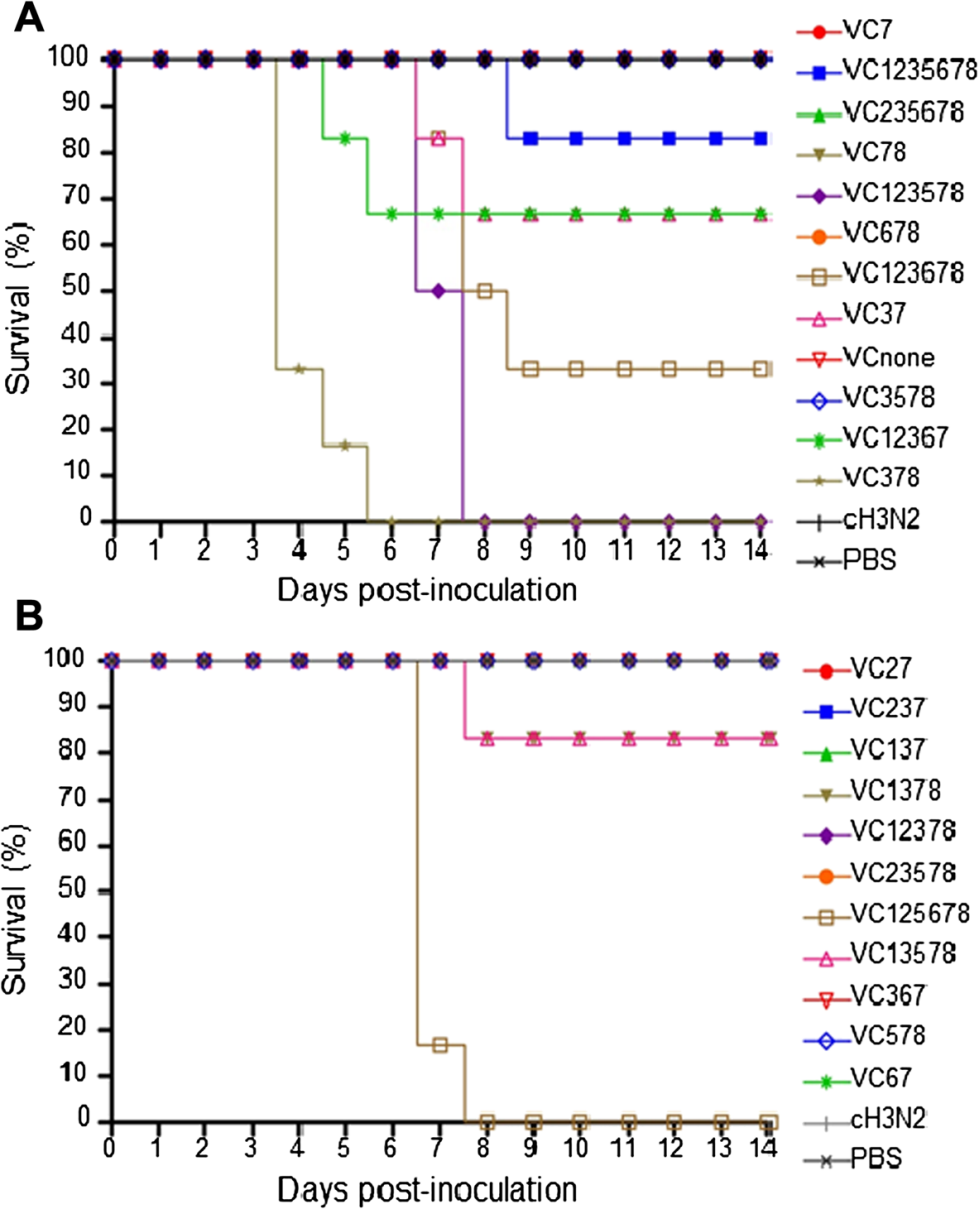
Survival rates of mice. Mice (*n* = 6 per group) were inoculated intranasally with 10^6.5^ EID_50_ of each virus or PBS and monitored daily for 14 days or until death. Panel **a** shows survival rates of groups of mice infected with VC7, VC1235678, VC235678, VC78, VC123578, VC678, VC123678, VC37, VCnone, VC3578, VC12367 or VC378. Panel **b** reveals those of groups of mice infected with VC27, VC237, VC137, VC1378, VC12378, VC23578, VC125678, VC13578, VC367, VC578 or VC67

For viral pathogenicity in mice, 6-week-old C57BL/6 mice (*n* = 6 per group) were intranasally inoculated with 10^6.5^ EID_50_ of virus. Mice were monitored daily for 14 days for changes in weight and mortality. The experiments conducted in animals were approved by an independent Animal Care and Use Committee and followed the guidelines of the Korea Research Institute of Bioscience and Biotechnology (Approval number 6015). In particular, mice infected with VC123578, VC378, or VC125678 showed severe clinical signs and experienced 100 % mortality within 8 days post-inoculation (Fig. 2). The mortality of other genotypes, namely, VC1235678, VC367, VC12367, VC37, VC123678, and VC13578 ranged from 17 to 67 %, while no morbidity or mortality was observed for the other genotypes including the original cH3N2 virus.

The routine surveillance for CIV by my colleagues has been conducted under the support of Ministry of Health and Welfare and Ministry of Science, ICT & Future Planning, Republic of Korea. In the present study, we isolated CIV reassortants from a dog that the co-infection of pH1N1 and cH3N2 viruses was confirmed through the surveillance as well as genetic recombination and analyzed the patterns of genetic recombination between cH3N2 and pH1N1 viruses. Collectively, the M gene of pH1N1 and the HA gene of cH3N2 are responsible for viral dominance, and this tendency was very similar to that reported in previous studies. For example, Ducatez et al. [6] reported multiple reassortants between pH1N1 and swine endemic influenza viruses; in particular, of seven distinct viral genotypes, each included the M gene from pH1N1 and 6, 5, 4, 3, 2, and 1 genotypes included the PA, NS, NP, PB1, PB2, and HA genes from pH1N1, respectively. The limited selection of the HA gene segment from pH1N1 was also consistent with that observed in previous studies involving the reassortants between pH1N1 and other influenza viruses [6, 12].

Genomic analysis of plaque-purified clones demonstrated 23 different genotypes of influenza viruses. All genotypes showed high infectivity by in vitro inoculation using MDCK cells or embryonated chicken eggs. However the genotypes displayed a broad spectrum of pathogenicity in vivo examination. Because multiple genetic exchanges had occurred in the clones, it is difficult to identify the reassortant of one or some gene segments that play a role in virulence in mice. Upon molecular analyses for the clones, pathogenicity-related point mutations (e.g., 591R or 591 K within the PB2 protein) were found in the gene segments from pH1N1 [13], although other known pathogenicity-related mutations (e.g., 158G, 627 K, and 701 N within the PB2 protein; 66S within the PB1 protein; and 97I within the PA protein) were not observed in the clones [14–16]. However, the mutations did not contribute to the differences in the pathogenicity of the genotypes observed during in vivo and in vitro examinations.

In our previous study in 2011, it was reported that a novel H3N1 CIV reassorted with pH1N1 and cH3N2 [8]. The isolate can be categorized under the VC1235678 genotype. Dogs inoculated with H3N1 CIV did not show notable clinical symptoms, while nasal virus shedding and mild histopathological lesions were observed following the experimental inoculation in host animals. Therefore, it needs to investigate virologic and pathologic examinations for CIV reassortants of the present study using animal models in further study.

In most industrialized countries, companion animals are an integral part of family life, sharing our lifestyles, bedrooms, and beds [17]. We had previously shown the possibility of natural reassortment between pH1N1 and cH3N2 in a dog. Thus, these results probably imply that a primary companion animal, which lives in closer proximity to humans than do pigs, might act as a mixing vessel or serve as a source of novel influenza A virus in humans. Furthermore, our findings emphasize that intensive monitoring for influenza infection in companion animals is necessary to investigate the potential for the emergence of novel human influenza strains.
